# Make it easier to be green: Solutions for a more sustainable planet

**DOI:** 10.1371/journal.pbio.3002064

**Published:** 2023-03-30

**Authors:** Andrew J. Tanentzap

**Affiliations:** 1 Ecosystems and Global Change Group, Department of Plant Sciences, University of Cambridge, Cambridge, United Kingdom; 2 Ecosystems and Global Change Group, School of the Environment, Trent University, Peterborough, Ontario, Canada

## Abstract

We urgently need solutions to make our use of the planet’s resources more sustainable and protect nature. A new collection of articles outlines a vision for a better tomorrow that draws on new advances in the development of green technologies.

You wouldn’t be mistaken for tuning out the constant hum in the background of our lives deploring the state of the natural environment and our ability to coexist with it. Things are indeed dire. Population growth continues to place ever-increasing demands on our planet’s limited resources, fostering a joint crisis of biodiversity loss and climate change [1]. Unsustainable practices in agriculture, transportation, and industry are contributing to mounting pollution and rising greenhouse gas emissions, pushing us beyond a safe space in which humanity can operate [2]. And warming temperatures, extreme weather, and rising sea levels are already affecting millions, largely unequally, with the Global South predicted to bear the brunt of these impacts [3]. But among the negativity, a new hope is rising. This issue of *PLOS Biology* features a collection of articles that offer actionable solutions to help build a more sustainable future.

Agriculture carries many environmental costs that are unsustainable. Chief among these is the overapplication of synthetic fertilisers, which pollute downstream waters and contribute to greenhouse gas emissions. While policy solutions exist to reduce synthetic fertiliser application and benefit the natural environment [4], a groundbreaking idea would be to leverage the natural associations that crops form with beneficial microbes to improve biological nitrogen fixation. In this collection, Jhu and Oldroyd [5] explore our emerging understanding of how legumes form symbiotic relationships with nitrogen-fixing plants. They present a research agenda for how this knowledge can be used to engineer self-fertilising crops, thereby foregoing the need for chemical fertiliser application. Another area where agriculture carries a large environmental footprint is related to the growth of crops for energy production—so-called “biofuels”. Many first-generation biofuels required massive agricultural areas that would displace food production [6]. This “food versus fuel” debate persists today. Cavelius and colleagues [6] provide an opinionated review of the current state of biofuels, highlighting the potential of genetic engineering to increase the production of desirable biomass-based energy sources, especially in microorganisms. Although they focus on the policy framework within the European Union, Cavelius and colleagues [6] offer clear actions to promote biofuels as a complementary technology for reducing anthropogenic greenhouse gas emissions.

The idea that microorganisms, in particular, can help solve many of our environmental problems is championed by many of the articles in this collection. A Perspective by Howe and Bombelli [7] asks if microbial photosynthesis has useful applications in directly generating energy. Biophotovoltaic devices have yet to demonstrate the potential to power small electronic devices in the real world, but Howe and Bombelli [7] outline the hurdles that need to be surpassed to realise their potential. Another Perspective by McCutcheon and Power [8] explores how microbes can directly capture atmospheric carbon dioxide and reduce the climate change impacts of emerging green energy solutions. Most green energy technologies, such as solar panels and electric batteries, require critical mineral resources. In their Perspective, McCutcheon and Power [8] highlight how the consumption of carbon dioxide by microbes growing in tailing ponds can turn mine waste into industrial carbon sinks while simultaneously extracting precious minerals to help meet rising global demands. Ralph [9] offers a related Perspective on the potential of algae to capture atmospheric carbon dioxide within manufacturing, such as in the beverage industry. Products manufactured using fixed carbon can add economic value to conventional carbon capture strategies currently touted by many governments worldwide [9].

The same principles of bioengineering solutions to our environmental problems are addressed in a set of papers by Bertocchini and Arias [10] and Ortiz [11], focusing on the environmental crisis caused by plastic pollution. Bertocchini and Arias [10] propose that rather than using microorganisms, newly discovered enzymes produced by insects can help degrade the most resistant synthetic polymers. This topic is truly an “Unsolved Mystery”, raising more questions than answers about how these enzymes work, where they exist in nature, and their potential to be harnessed by biotechnology solutions. Rather than treat the symptom of excessive synthetic plastic production, Ortiz [11] provides a firsthand Perspective on pioneering work to develop plastics from renewable biological sources. Although the hope is that these bioplastics will degrade more easily in the environment, their environmental impacts remain an open question.

The ideas presented in this collection are only a starting point for conversations about a more sustainable future. Many more solutions exist than we could cover in this collection, so this set is not meant to be exhaustive or definitive. However, irrespective of the promise of technology, environmental impacts at all stages of a product’s life cycle must be assessed formally and form the foundation of any attempts to embrace new solutions. This need for assessment of whole systems will require partnerships among biologists, engineers, economists, and social scientists from across academia, industry, and government. A wider greener “revolution” for a more sustainable future is ahead of us, and we hope that this collection inspires and informs progress towards this goal.
